# Response Systems, Antagonistic Responses, and the Behavioral Repertoire

**DOI:** 10.3389/fnbeh.2021.778420

**Published:** 2022-01-13

**Authors:** Daniele Ortu, Ryan M. Bugg

**Affiliations:** Neurobehavioral EEG Laboratory, Department of Behavior Analysis, University of North Texas, Denton, TX, United States

**Keywords:** response systems, behavioral repertoire, response competition, anatomical constraints, basal ganglia

## Abstract

While response systems are often mentioned in the behavioral and physiological literature, an explicit discussion of what response systems are is lacking. Here we argue that response systems can be understood as an interaction between anatomically constrained behavioral topographies occasioned by currently present stimuli and a history of reinforcement. “New” response systems can develop during the lifetime as the organism gains instrumental control of new fine-grained topographies. Within this framework, antagonistic responses compete within each response system based on environmental stimulation, and competition is resolved at the striatum-thalamo-cortical loops level. While response systems can be by definition independent from one another, separate systems are often recruited at the same time to engage in complex responses, which themselves may be selected by reinforcement as functional units.

## The Repertoire: Single, Independent, and Coordinated Response Systems

Instrumental conditioning provides a thoroughgoing account of how complex environment-behavior relations develop, and may be useful to neuroscientists as a framework for understanding the neurophysiological processes and mechanisms involved in the development of the behavioral repertoire (e.g., Donahoe et al., 1993). Comparing the behavior of an adult human to that of an infant or child makes clear the role of ontogenetic selection in the development of response systems. The human infant is capable at first of only gross motor responses. These “primordial” responses are differentiated over time into successively finer-grained responses, eventually permitting the full range of human conduct, or repertoire, from catching a ball, speaking in sentences, to driving a car—or any other behavioral topography available to the adult human.

But what is the behavioral repertoire in the first place? The repertoire is a construct referring to the behavior an organism is capable of as a result of both its phylogenetic and ontogenetic histories. Under certain conditions, one response is more probable than others due to a particular history of behaving under similar conditions (e.g., Catania, 2013). Over time, the experienced organism becomes capable of engaging in a wide-range of behavior under an equally wide-range of environmental conditions. As such, this large number of possible behavioral responses must be coordinated to avoid the occurrence of chaotic behavior. To understand how this takes place, a neurobehavioral conceptualization of response systems may be of pragmatic value.^1^ Our take on response systems is a preliminary effort focusing mostly on environmental cues in relation to responses and response systems, and not on the vital role of reinforcing stimuli in organizing response systems according to ethologically relevant categories (e.g., Graziano, 2006). Moreover, the current paper does not extensively address the complexities involved in rapid behavioral changes due to shifting contingencies; a relevant issue when an outcome following a specific action changes in value and the remote and recent history of learning interact.

A response system may be considered an anatomically constrained and defined subset of environment-behavior relations. Environment-behavior relations in turn are conceptualized here as cues increasing or decreasing the probability of certain behavior, based on previous instances of reinforcement and/or punishment when that behavior occurred in presence of those stimuli (for a review, see Dinsmoor, 1995a,b). A microwave oven emits a beeping sound, a person approaches the oven, opens its door, and retrieves a meal. But what about instances in which multiple activity patterns simultaneously occur? For instance, a skilled individual may be able to ride a bike, play guitar and sing at the same time. How does this take place?

Before tackling the complexities involved in multiple independent and coordinated response systems, let us then start from the assumption that within an individual response system, only one response occurs at any given time. Along those lines, Palmer (2009) mentions the following:

In any one response system, most responses are mutually incompatible. We can walk to the left of a tree or to the right of the tree, but we can’t do both. We can say, “Hello,” or “Hi there,” but not both, at least not at the same time. Response competition is an important concept. If we tried to engage in every response that had some strength, our behavior would be chaotic. If two incompatible responses are roughly equipotent, only one will actually be emitted. The competing response appears to be inhibited (p. 56).

**FIGURE 1:**
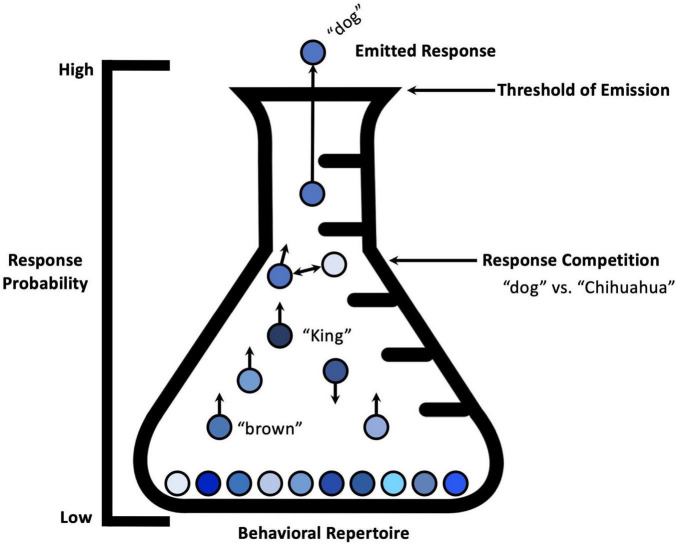
Emission of a single response belonging to a single response system. A visual metaphor showing the emission of a single response, and the relative changes in response strength of responses within the same response system. Specific environmental conditions occasion a specific behavioral response, despite multiple members of the response class being suitable for a given situation. Response competition is characterized by bidirectional arrows between the particular responses within the response system. Adapted from “Response strength and the concept of the repertoire,” by Palmer (2009).

According to this conceptualization, within each response system, the strength of a response varies in a moment-to-moment fashion as a function of current environmental stimulation and an organism’s learning history. Considering the multitude of behaviorally relevant stimuli encountered by an organism at any given moment, an important question is raised: What prevents multiple responses from occurring at the same time and potentially resulting in a maladaptive response topography? Let us consider Palmer’s example of a person trying to go both left and right at the same time. Palmer suggests that at any given moment, the probability of emission for each response in the behavioral repertoire is influenced by current environmental stimulation: “shifts in stimulus control can favor the target response so that it becomes the dominant response in its response system” (p. 51). In other articles, behavioral scientists describe how current environmental stimulation potentiates a variety of responses within a given response system (e.g., Michael et al., 2011). For example, the production of the sounds making up vocal responses involves the coordination of a response system of facial and vocal tract muscles, as well as muscles belonging to the diaphragm and other components of the respiratory system. Given the anatomical organization of the human vocal apparatus as it is supported by thalamo-cortico-striatal circuitry, humans seem able to engage in a single vocal response at any given time (see Figure 1). Michael et al. (2011) elucidate this phenomenon:

When we see a dog, we cannot simultaneously say, dog, King, brown, Chihuahua, etc. [.]All behavior within a response system can be thought of as in competition with other behavior in that response system. Thus, many verbal responses may be relatively strong at a particular moment, but only one can be emitted at a time. Presumably one response, the prepotent response, is stronger because of its conditioning history, or perhaps because of the confluence of other evocative variables at the moment (p. 7).

Current stimulation, therefore, changes the probability of occurrence of many responses, as evidenced by a large number of priming experiments (e.g., Neely, 1991; for a behavior analytic interpretation see Palmer, 2009; Ortu, 2012). In a priming experiment, a priming stimulus is presented (for instance, the word “coffee”) and is either followed by a “related” target stimulus (e.g., “milk”) or an “unrelated” target stimulus (e.g., dog). In these experiments, carried out typically with a large number of trials, participants are faster in emitting a textual response after the “related” target compared to the unrelated target. The implication of these studies is that the presentation of the priming stimulus changes the response strength, or probability of emission, of a large number of potentially antagonistic responses.

**FIGURE 2:**
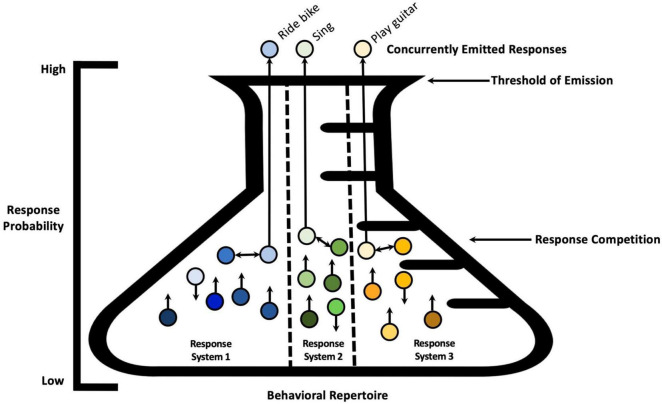
Emission of three responses at a given time belonging to three separate response systems. A visual metaphor showing three response systems within the behavioral repertoire, and the simultaneous emission of three behavioral responses belonging to three separate response systems. Antagonistic responses within each response system are noted with bidirectional arrows between the responses. Since these responses belong to separate response systems, the individual can learn to emit them at the same time, as there are no anatomical limitations preventing them.

The notion of response antagonism, or competition, might be expanded to account for multiple response systems (see Figure 2). For instance, if current textual stimulation (e.g., Starbucks, Latte, Cappuccino) increases the strength of the vocal response *coffee*, leading to the emission of a vocal response ‘‘coffee,’’ emission of the vocal response should not prevent the organism from engaging concurrently in behavior involving another response system, for example, walking^2^ (Palmer, 2009; for an alternative interpretation based on the spreading activation see also Klinger et al., 2000). Anatomically, the vocal apparatus and the locomotive apparatus appear relatively independent. Anatomy, then, imposes the boundaries within which ontogenetic development occurs; thus, response system organization develops from the interaction of anatomical constraints and learning variables (e.g., reinforcement learning/selection by consequences). As another example, given the anatomical organization of human arms and hands, multiple responses can be emitted concurrently; and within each hand, each finger can potentially respond independently from the others, provided the organism has learned to do so. Additionally, consider the hypothetical example of an individual born with six fingers on each hand learning how to play the piano. This person would have access to *topographic degrees of freedom* unavailable to a person born with five fingers on each hand. If we conceptualize each finger’s movement as a new potentially independent response system, the addition of the sixth finger may add a new response system—a new “partition” within the overall repertoire.

Consider another example from the musical domain: Great drummers are known for acquiring complete limb independence. While reading a score or improvising, the responses of each limb are effectively independent of the others. This can be probably demonstrated best in an instance of sight-reading with a score the drummer has not seen before. Ultimately, however, anatomy never ceases to provide constraints. Even when full limb independence is gained, a drummer with especially long arms or legs may be able to execute parts unachievable to a drummer with shorter limbs, and vice-versa. This is also relatively evident in sports: A very tall basketball player may have access to topographies unavailable to a relatively short player, and vice-versa. Going back to the priming phenomenon, given that organisms are constantly “bombarded” by behaviorally relevant stimuli, and that the same stimulus may divergently affect *different* response systems, a further implication is suggested: At any given moment there may be as many instances of response competition as there are response systems.

As a corollary, if anatomical constraints are (at least in part) what is driving the separation across response systems, it is possible in principle that if those anatomical constraints were eliminated, then the organism—given the appropriate training—could learn to engage in a number of concurrent responses that would in principle be constrained only by the computational capabilities of the brain. Brain-Machine Interface (BMI) research has recently highlighted how monkeys with implanted electrodes in their brain cortex can learn to control additional robotic limbs (Carmena et al., 2003). Analogously, from a stimulus perspective, rats have been shown to be able to respond to changes in infrared light when an external sensor is connected to their brain cortex (Thomson et al., 2013), while concurrently discriminating other changes in the visible light spectrum. The same neural populations appear able to simultaneously “learn” how to respond to multiple sources of stimulation, incoming from both biological and electronic receptors.

Given the possibility of n-number of additional non-biological interfaces to operate on the environment, and n-number of additional receptive mechanisms to detect incoming stimulation, the behaving capabilities of organisms appear potentially expandable beyond the current limitations dictated by the anatomical constraints due to natural selection. Organisms are at birth equipped with response systems that were congenial at points in time that may not reflect current environments. The possibility of adding response systems (e.g., robotic arms) and stimulus systems (e.g., robotic eyes) may greatly expand the behaving potential of organisms. Moreover, both response and stimulus systems need not be physically connected or located in the same environment as the biological organism. Response and stimulus systems can potentially be located in additional environments, with the biological organism constituting the physical locus, or hub, in which the actual, mechanistic selection of environment-behavior selection takes place at the neural level.

Whether or not response and stimulus systems are biological, we must consider both the potential gain of independence among response systems, as well as coordination among the partitions. When a person must jump suddenly to the right to avoid a ball thrown at him/her, an ensemble of potentially independent response systems is recruited at the same time, including leg muscles, postural muscles in the back, neck muscles, and others. How does this concurrent recruitment of potentially independent response systems occur, and happen so quickly? Is the coordinated response selected as a single functional unit, occasioned by specific stimulus conditions that differ from the ones that evoke responses from individual response systems? This leads us to another pressing concern: If these coordinated efforts involving multiple response systems are selected as units, i.e., a complex response unit,^3^ is it the case that other, incompatible, complex response units are inhibited in the same way as they are within an individual response system when a dominant response has acquired critical strength? The behavioral account may be informed by the current understanding of the neural mechanisms related to these changes in probability and subsequent emission of a particular response over others. Evidence at the thalamo-cortical-striatal level in particular may illuminate behavioral descriptions, especially “winner-takes-all” models of basal ganglia function.

## Neural Mechanisms Supporting Response Competition and Emission Across Response Systems

A review of the neuroanatomical literature, and especially of the topographically organized loops at the thalamo-cortical-striatal level, suggests a possible boundary-bridging convergence of the behavioral and neurological levels of analysis (e.g., Abrahamsen, 1987; Ortu and Vaidya, 2016). For instance, the continual restructuring of response systems at the behavioral level parallels selectionist accounts of the nervous system and the formation of cortical maps (Edelman, 1993). While the central nervous system facilitates the coordination of various effector systems, behavioral selection provides an ultimate cause for the organization of response systems making up the repertoire (Donahoe and Palmer, 1994).

From a response-outcome perspective, experiments have shown that the posterior dorsomedial striatum (pDMS) is involved in supporting rapid learning due to shifting environmental contingencies caused by changes in outcome value (Balleine and O’doherty, 2010; Shiflett et al., 2010). More specifically, the parafascicular thalamic nucleus (PF) is connected to the pDSM and contributes (via cholinergic interneurons) to selecting responses when a remote learning history “conflicts” with recent changes in outcome value (Bradfield et al., 2013).

From a stimulus-response perspective, the basal ganglia, the motor cortices, and the thalamus are involved in a neural loop critical in inhibiting competing responses once a dominant response has reached a critical point of “no return” (e.g., Middleton and Strick, 2000; Silkis, 2000, 2001; Haber and Mcfarland, 2001; McHaffie et al., 2005; Hayhow et al., 2013; Oldenburg and Sabatini, 2015). More specifically, the literature describes two co-active and interactive pathways—the direct and indirect—generally considered to be involved in response inhibition and selection (Hong and Hikosaka, 2011; Tecuapetla et al., 2016).

The thalamus plays a central role in these pathways due to its polymodal inputs from the sensory cortices and outputs to motor cortices. Further, it receives inhibitory GABAergic inputs from two striatal areas within the basal ganglia: the internal segment of the globus pallidus and the substantia nigra pars reticulata^4^ (e.g., Hikosaka, 2007, see also McElvain et al., 2021). This interaction suppresses downstream initiation of movement by reducing overall thalamic activity (Plenz, 2003). Additionally, the striatum receives excitatory inputs from the cortex, resulting in the release of glutamate, and the subsequent activation of GABAergic neurons in the striatum. This release of GABA inhibits activity in the striatum, thus disinhibiting thalamic neurons and allowing the occurrence of a motor response (Chevalier and Deniau, 1990).

The indirect pathway has an equal role in inhibiting and disinhibiting motor responses. Within the basal ganglia, GABAergic neurons project from the globus pallidus and inhibit the neurons of the subthalamic nucleus, modulating striatal activity and broadly altering the interactions of the direct pathway. The indirect pathway is also at times activated by projections from the cortex inhibiting neurons in the globus pallidus. As a result, the activity of subthalamic neurons increases, and thus the inhibitory effect of the basal ganglia also increases resulting from the release of GABA. More recent research has proposed an additional “hyperdirect” pathway from cortical areas to the subthalamic nucleus (Nambu et al., 2000, 2002; Milardi et al., 2019; Chen et al., 2020). These mechanisms ultimately support the emission of responses via activation of neurons in motor areas^5^.

Feedforward relations between premotor and supplementary motor areas eventually activate neurons in primary areas that are individually tuned in a very specific way to many motor features, similar to sensory-perceptual neurons in the IT cortex and their fine-tuned sensitivity to a large number of stimulus features (Desimone et al., 1984). Moreover, the classic idea that neurons in the primary motor cortex are organized in a one-to-one mapping with muscle effectors has been recently challenged by a more complex perspective involving the concept of many-to-many mapping. The many-to-many theory (e.g., Graziano, 2006; Huber et al., 2020) posits that individual neurons in primary motor areas can affect many different muscle effectors and that each effector can be controlled by many neurons in motor areas. From a response system perspective, this complexity allows for the dynamic and plastic recruitment of populations of neurons based on ethologically and behaviorally relevant categories.^6^ These categories are not fixed but are repeatedly updated based on the feedforward and feedback interactions of dopaminergic signals from the ventral tegmental area and the substantia nigra to the dorsolateral striatum (e.g., Graybiel, 1990).

In conclusion, activation of effectors involved in a response system may then require the interaction between the basal ganglia and motor areas in concurrently inhibiting populations of neurons involved in competing responses and disinhibiting populations of neurons involved in the dominant response, based on current stimulation. Given the many-to-many mapping between neurons in motor areas and effectors, this population coding (Georgopoulos et al., 1986) may involve overlapping sets of neurons in motor areas in dominant and competing responses within a response system. When anatomical constraints are absent, multiple movements may occur concurrently due to the co-activation of multiple response systems. Under these conditions, the basal ganglia-motor cortex interaction may allow disinhibition—based on previous learning—of many populations of neurons coding for non-conflicting responses.

## Data Availability Statement

The original contributions presented in the study are included in the article/supplementary material, further inquiries can be directed to the corresponding author/s.

## Author Contributions

DO wrote a first preliminary draft of the manuscript. RB contributed to all contents and sections in a deep and fundamental way. Both authors contributed to the article and approved the submitted version.

## Conflict of Interest

The authors declare that the research was conducted in the absence of any commercial or financial relationships that could be construed as a potential conflict of interest.

## Publisher’s Note

All claims expressed in this article are solely those of the authors and do not necessarily represent those of their affiliated organizations, or those of the publisher, the editors and the reviewers. Any product that may be evaluated in this article, or claim that may be made by its manufacturer, is not guaranteed or endorsed by the publisher.

## Footnotes

1 The organization of behavior provides a framework to analyze neurophysiological events by defining the boundaries within which relevant physiological mechanisms operate (Donahoe and Palmer, 1994; Krakauer et al., 2017).

2 In subvocal speech, a person is typically not able to engage in multiple concurrent vocal streams: It appears that the constraints typical of the vocal apparatus are maintained in subvocal speech. When the vocal response becomes subvocal through ontogenic development, what prevents individuals from learning to engage in multiple parallel vocal streams? The subvocal, or covert, version of the performance of interest also appears to be constrained by anatomical factors even when considering other behaviors. For instance, a guitar player who is covertly rehearsing a scale will not do so by using an arbitrary number of fingers and arms. Rather, the anatomical constraints that were present during learning still play a role in future covert practices. Similarly, when a person loses a limb or loses the ability to control some or all muscles due to paralysis or amyotrophic lateral sclerosis (ALS or Lou Gehrig’s disease), they still appear to be able to retain covert speech and other covert topographies.

3 Response complexity refers to both the diachronous emission of responses making up a particular motor program (see Jin et al., 2014 for a discussion of response “chunking”), as well as the synchronous emission of multiple responses (e.g., such as when a piano player learns to play one chord with the left hand and another with the right at the same time) unified into a single behavioral response pattern. Added to this consideration, responses of differing complexity may be evoked by stimuli that also vary in complexity. An individual relatively simple stimulus (e.g., a square) can be embedded within a simple context (e.g., a white background); a complex stimulus (e.g., a human face) can be embedded in a simple context or a complex context (e.g., a specific configuration of contextual stimuli). Stimuli—both objects and contexts—can also vary in complexity over time (e.g., a stimulus may morph from simple to complex within 5 s and at the same time the context can shift from complex to simple), and responses can potentially track these stimulus changes.

4 The SNr and GPi also send GABAergic projections to a variety of other nuclei including the Superior Colliculus and the Pedunculupontine Nucleus.

5 It is important to acknowledge that research on neural mechanisms involved in response competition is still partially speculative and based on physiologically informed computational models (e.g., Baston and Ursino, 2015).

6 Ethologically and behaviorally relevant stimulus categories are coded by different subpopulations of the amygdaloid complex (e.g., Pignatelli and Beyeler, 2019).
